# The perceived competency and attitude of emergency nurses towards the patients boarding in ED with psychiatric illness: A cross-sectional study in Qatar

**DOI:** 10.5339/qmj.2026.1

**Published:** 2026-03-03

**Authors:** Nesiya Hassan, Hashem A. R. Mestarihi, Ameneh Ahmad Zadeh, Bejoy Chacko, Grethel Dela Paz, Rajvir Singh

**Affiliations:** 1 Nursing and Midwifery Research Department, Hamad Medical Corporation, Doha, Qatar; 2 Nursing and Midwifery Department, Hamad General Hospital, Hamad Medical Corporation, Doha, Qatar.; 3 Academic Health System, Hamad Medical Corporation, Doha, Qatar *Email: nesiyahassan@gmail.com

**Keywords:** Clinical competence, attitude, Emergency Medical Services, Psychiatric Emergency Service, mental disorders

## Abstract

**Introduction:**

The boarding of patients with psychiatric illnesses in the Emergency Department (ED) has become a prevalent issue across the world, posing significant challenges for healthcare providers.

**Objectives:**

This study aimed to assess the perceived competency and attitude of emergency nurses toward the care of patients boarding with psychiatric illnesses.

**Methodology:**

This was a prospective cross-sectional research study used to recruit emergency nurses working in four selected facilities under the organization. The study utilized a validated questionnaire, the Opening Minds Scale for Health Care Providers (OMS-HC-15) and Behavioral Health Care Competency (BHCC) to collect the information from the participants through an online survey using a total enumerative sampling method. Frequency and percentage were used to find the demographic characteristics of the participants. ANOVA and t-tests were used to find out the association between the attitudes, competencies, and demographic profile of the participants.

**Results:**

Three hundred and ten completed responses from the participants were used for analysis. Most participants were in the 31 to 40 years age group (72.26%; *n* = 224), with a mean age of 42 years, which ranged from 26 to 56 years. Males (*n* = 183) participated more compared to females (*n* = 127), and 85.16% (*n* = 264) of them have Bachelor’s degrees in nursing. 40% (*n* = 124) of the participants have more than 10 years of experience in ED. The mean percentage scores of attitude and competency domains were −14.16 ± 36.26 and 35.07 ± 25.73, respectively. The competency domain showed a statistically significant correlation with the participants’ age (P = 0.038) and their facility (P = 0.014). In contrast, attitude did not exhibit statistical significance with any demographic variables.

**Conclusion:**

The emerging data from the current study stated that the attitudes of ED nurses were predominantly positive; however, their perceived competency was falling below average. This highlights the need to improve their competency to strengthen the current practice, the quality of care, and the overall experiences of psychiatric patients in the ED.

## 1. INTRODUCTION

The importance of mental health has reached the attention at the global level. The World Health Organization (WHO) illustrated that mental health is an integral part of the Sustainable Development Goals and estimated the prevalence of mental disorders was 13% globally.^1^ The consequences of this problem may extend beyond individual suffering, impacting social and public health concerns.^2^ Addressing these discriminations is critical in healthcare settings for ensuring the dignity, respect, high-quality care, and well-being of psychiatric patients.^2^ As the demand for psychiatric patient care in Qatar continues to increase, the Emergency Department (ED) healthcare team faces many challenges in providing high-quality care to these patients. Healthcare workers’ attitudes towards psychiatric patients can affect the quality of care and health outcomes.

The boarding of patients with psychiatric illnesses in EDs has emerged as a serious issue globally, challenging healthcare systems as well as providers.^3^ Recent research findings indicate that around 10% of the entire ED patient population consists of individuals with psychiatric conditions.^4^ Mental health patient assessment and triage decisions can be challenging for ED nurses due to the noisy environment and lack of privacy where these assessments are conducted.^5^ The prolonged stay of psychiatric patients in the ED due to the unavailability of appropriate inpatient psychiatric beds leads to overcrowding, compromised patient care, increased healthcare costs, and heightened emotional distress for both patients and healthcare providers.^6^ Patients experiencing psychiatric challenges frequently seek care in the ED during periods of crisis.

A study from the Middle East revealed that the nurses had positive attitudes regarding the rights of patients, including the right to receive a safe environment, the right to select the health care institution, and the right to benefit from healthcare services according to their medical condition.^7^ A specialized training in mental health and personal contact with patients suffering mental health illness were important predictors of stigma and knowledge.^8^ These findings were supported by Granados-Gámez et al.,^9^ However, the participants of another study reported negative attitudes toward mentally ill patients.^10^ The inconsistency in nurses’ attitudes reflects varying levels of acceptance and understanding of mentally ill patients. Qatar is a rapidly developing country with a diverse population and faces challenges in its healthcare system, such as insufficient training and low motivation among the primary care workforce, a shortage of mental health services and professionals, and a higher level of stigma that discourages individuals from seeking mental healthcare.^11^ The national health strategies in Qatar 2018 to 2022 provide key priorities in improving access to safe and high-quality mental health services for people living in Qatar.^12^ Despite of significant impact of overcrowding in ED with boarding psychiatric patients, there is a lack of research to assess the emergency nurses’ perceived competency and attitude of ED nurses toward caring for such patients. ED nurses are specialized and competent in managing emergency-based care of patients with complex medical and trauma care. However, they may lack competencies in de-escalation techniques and crisis communication, leading to a lack of confidence in intervening in situations that require specific skills.^13^ Additionally, the evaluation and treatment of psychiatric patients in the ED may not always follow in line with the treatment received in psychiatric inpatient units. For instance, where patients are acutely agitated, hostile, aggressive, or suicidal, it may be necessary to rapidly treat their psychiatric symptoms with medications before a full workup is completed.^14^ This highlights the unique and different challenges that psychiatric patients present in the ED. Nurses working in non-psychiatric units, especially in ED, may face challenges in caring for patients with psychiatric behaviors and may lack the necessary confidence and competencies for managing such situations. In this context, understanding the nurses’ attitudes and competency is essential for developing effective strategies to address the unique needs of such patients. This study seeks to bridge the knowledge gap and contribute valuable insights to the field of emergency nursing and psychiatric care in Qatar. This cross-sectional study aimed to assess the perceived competency and attitude of emergency nurses regarding the care of patients with psychiatric illnesses in the ED.

## 2. METHODOLOGY

### 2.1 Study design

This study used a prospective cross-sectional research design to assess the perceived competency and attitude of emergency nurses toward caring for patients admitted to the ED with psychiatric illnesses.

### 2.2 Study settings

The study was conducted among the emergency nurses working in four major public hospitals in Qatar, including Al Wakrah Hospital (AWH), Al-Khor Hospital (AKH), Hamad General Hospital (HGH), and Hazm Mebaireek General Hospital (HMGH) under Hamad Medical Corporation. The organization consists of 14 healthcare facilities that render medical care for citizens as well as residents of the country. The total bed capacity of these four EDs is 360, with 14 beds dedicated to psychiatric patients’ admission.

### 2.3 Time period of study

The study was conducted for approximately 1 month from December 27, 2023, to January 16, 2024.

### 2.4 Sampling technique

The organization employs approximately 10,000 clinical nurses across different facilities. The study population consists of 820 ED nurses working in four health care facilities. A total enumerative sampling method was utilized to collect data.

### 2.5 Sample size

This study population consists of 820 emergency nurses, which includes 150 registered nurses (RNs) in AWH, 75 in AKH, 515 in HGH, and 80 in HMGH. The 820 emergency nurses were screened for eligibility criteria, and the author considered the 50% rule to calculate the sample size. The study required 262 emergency nurses at a 95% confidence interval and 5% precision, and with the consideration of a 10% incomplete questionnaire, the final sample size was 288.^15^ However, 310 completed responses received during the index period were taken for final analysis. The sample size was determined using the online Sample Size Calculator available at Calculator.net.^16^

### 2.6 Inclusion criteria

The study included licensed emergency nurses with a minimum of 1 year of experience in the selected ED during the index period.

### 2.7 Exclusion criteria

Nurses without one annual performance appraisal and those who are not involved in direct patient care were excluded from the study.

### 2.8 Data collection procedure

The data were collected from the participants through an online survey. The list of eligible participants was collected from the nursing administration of each hospital. The participants were invited to the study through an open invitation in their official email, along with the research information sheet and a link to the survey. Anonymity and confidentiality of the participants were maintained in this study. The identity of the participants was not collected at any stage of the study. The survey closed when the required responses were reached. The Microsoft form was used to collect data from the participants.

### 2.9 Data collection instrument

The study collected the information by using two validated questionnaires: The Opening Minds Scale for Health Care Providers (OMS-HC-15) and Behavioral Health Care Competency (BHCC). The demographic part collected information regarding age, gender, educational status, experience in ED, experience in caring for aggressive patients, and various training statuses.

The OMS-HC-15 was a self-reported questionnaire developed by Modgill et al.^17^ The OMS-HC-15 consists of three subscales: (1) Attitudes of health care providers towards people with mental illness (six items); (2) Disclosure/help-seeking (four items); and (3) Social Distance (five items). This study uses only the attitude subscale with six items, with a maximum score of 12. The attitude subscale uses a five-point Likert scale with options from strongly disagree to strongly agree. The OMS-HC-15 has an overall internal consistency of α = 0.79, and the internal consistency of each subscale (Attitude, Disclosure and Help-seeking, and Social Distance) is above 0.65 with good test–retest reliability (attitude: r = 0.82, disclosure: r = 0.73, social distance: r = 0.74). None of these items required reverse coding.^18^

The BHCC was developed by Rutledge et al.^19^ The BHCC scale consists of 23 items with four subscales: Resource Adequacy (four items), Patient Assessment (nine items), Practice/Intervention Competency (eight items), and Psychotropic Recommendation (two items). The total internal consistency of the BHCC Cronbach’s alpha was 0.91, with each subscale demonstrating adequate internal consistency assessment (α = 0.85), practice/intervention competency (α = 0.85), recommendation of psychotropics (α = 0.79), and resource adequacy (α = 0.69). The 23-item scale has a total score of 46.

In both scales, the author coded a five-level Likert scale as "strongly disagree", "slightly disagree", "neutral", "slightly agree" and "strongly agree", as "−2", "−1", "0", "+1" and "+2" respectively.^20^ The formula ∑(items response)/∑(highest values in the items) was used to determine the individual index for the attitude and competency domain.^21^ The mean percentage score was calculated by multiplying each domain index score by 100. In this cross-sectional study, the data were collected from the participants through an online survey using Microsoft Forms, and the data were transferred to Microsoft Excel for analysis.

### 2.10 Data analysis technique

The sample characteristics were summarized and analyzed using descriptive statistics. Data that followed a normal distribution were presented using the mean and standard deviation. Frequency and percentage were used to find the demographic profiles of emergency nurses in terms of age, gender, education, ED experience, specialty experience in psychiatry, training including management of assaultive behavior (MAB), suicide prevention, behavioral and mental status assessment, etc.

The attitudes of the emergency nurses were calculated by using six items from the OMS-HC-15 questionnaire. The competencies of the emergency nurses were computed using 23 items from the BHCC scale. To analyze the associations of the attitudes, competencies, and demographic profile of the emergency nurses (age, gender, education, experience, experience in psychiatry specialty, training including MAB, suicide prevention, behavioral and mental status assessment), ANOVA and t-tests were used. The formula ∑(items’ response)/∑(highest values in the items) was used to determine the individual index for the attitude and competency domain.^21^ The mean percentage score was calculated by multiplying each domain index score by 100. All P values presented will be two-tailed, and P values <0.05 will be considered statistically significant. All statistical analyses will be done using the statistical package SPSS, version 29.0.

### 2.11 Ethical approval

The research was carried out in strict adherence to the “Declaration of Helsinki” principles, Good Clinical Practice (GCP) guidelines, and in accordance with the laws and regulations of the Ministry of Public Health (MoPH) in Qatar. Ethical approval was obtained from the IRB of the Medical Research Center (MRC), protocol # MRC-01-23-771 on December 24, 2023.

## 3. RESULTS

### 3.1 Sample characteristics

The survey was sent to 820 nurses working in selected EDs during the index period. Three hundred and forty-five responses (response rate = 42.07%) were received from the participants, and 35 incomplete survey responses were excluded. A total of 310 completed responses were used for the final analysis. The participant’s sociodemographic characteristics are displayed in Table 1. The 31 to 40 years age group was most represented (72.26%), followed by the age group of 41 to 50 years (18.06%). The mean age was 42 years, which ranged from 26 to 57 years. The male-to-female ratio was 1:1.4, and 85.16% of them have a minimum of a bachelor’s degree in nursing, and 7.42% hold a master’s degree in nursing. Forty percent of the participants (*n* = 124) have more than 10 years of experience, followed by less than 5 years and 6 to 10 years of experience, which account for 30.97% and 29.03%, respectively. Most of the participants (69.03%) had no prior experience in a psychiatric specialty, whereas the majority had experience in caring for aggressive patients in the ED (80.65%). Furthermore, most respondents reported a lack of training in essential skills, including assaultive behavior management (54.19%), suicide prevention (83.87%), behavioral therapy (84.19%), and mental status assessment (69.58%).

### 3.2 The perceived competency and attitude of ED nurses

Table 2 illustrates the attitude and competency of the participants towards patients boarding at the ED with mental illness. The calculated mean percentage of attitude was −14.16 ± 36.26. By subtracting the mean score percentage from 100, the attitude was 86%, which indicates the participants have a positive attitude towards caring for patients with mental illnesses. Similarly, the calculated competency mean percentage was 35.07 ± 25.73, indicating a 35% competency toward caring for such patients. Within the competency subdomains, the highest mean percentage score was in the resource subdomain (45.52 ± 29.99), followed by assessment (39.55 ± 26.02). The practice and recommendation of psychotropic medications scored lower, with mean percentages of 30.40 ± 32.18 and 12.74 ± 46.37, respectively.

### 3.3 The factors associated with perceived BHC competency and attitude of ED nurses

Table 3 illustrates the association between participants’ age, gender, qualifications, experience, and assigned facility with their attitudes and BHC competence. The attitude domain showed no statistical association with age, gender, educational status, years of experience, or the facility of the participants. Whereas the BHC competency shows a statistically significant association with facility (0.383 ± 0.233; P = 0.014) and age (0.502 ± 0.286; P = 0.038) of the participants.

#### 3.3.1 Association with the BHCC subdomain and age of the participants

The age of the participants shows a statistically significant association with the subdomain practice; the 21 to 30 years age group had a higher mean score (0.489 ± 0.356; P = 0.015) compared with other age categories, including 31 to 40 years (0.325 ± 0.298), 41 to 50 years (0.214 ± 0.367) and >51 years age group (0.214 ± 0.372).

#### 3.3.2 Association with BHCC subdomain and gender

The variable gender had a statistically significant association with practice, recommendation, and resource subdomains of the BHC competency. The male participants hold higher mean scores compared with females, in subdomains practice (0.377 ± 0.293; P = 0.002), Recommend (0.231 ± 0.421; P = 0.033), and Resource (0.492 ± 0.272; P = 0.004).

#### 3.3.3 Association with the BHCC subdomain and the experience of the participants

The experience of the participants has a statistically significant association with practice and resource subdomains. The practice subdomain holds a mean score of 0.365 ± 0.291 (P = 0.025) in the <5 years’ experience group and compared with 6 to 10 years (0.311 ± 0.293) and >11 years age (0.247 ± 0.352) groups.

#### 3.3.4 Association with the BHCC subdomain and the facility of the participants

Interestingly, facility C shows a statistically significant association with BHC competency (0.383 ± 0.233; P = 0.014) compared with other facilities. A similar pattern continues with the subdomains assessment (0.425 ± 0.238; P = 0.028), recommendation (0.181 ± 0.445; P = 0.048), and resource (0.493 ± 0.281; P = 0.014) subdomains compared with other facilities.

### 3.4 The association between training and experience with competency and attitude

Table 4 illustrates the association between various training courses, experience in psychiatric specialty, BHC competency, and the attitude of ED nurses. The participant’s experience in the psychiatric specialty (−0.160 ± 0.416; P = 0.013), and training in the Management of Assaultive Behavior (−0.150 ± 0.393; P = 0.046) had a statistically significant association with the attitude domain compared with those nurses without experience in the psychiatric specialty (−0.134 ± 0.337) and MAB (−0.135 ± 0.336). Experience in handling aggressive patients (P = 0.17; P = 0.05), suicide prevention training (P ≥ 0.99; P = 0.85), behavioral therapy (P = 0.33; P = 0.66), and mental status assessment (P = 0.27; P = 0.42) showed no statistically significant difference with the attitude and competency domains of the participants. These findings indicate that experiences in caring for psychiatric patients and training in managing assaultive behaviors contribute to a positive attitude among ED nurses. However, any of these factors alone is sufficient to enhance their competency. Table 5 illustrates the correlation between the attitude and competency of ED nurses. The Pearson Correlation shows that the relationship between attitude and competency is not significant, r = −0.065; P = 0.25, indicating that both are independent variables.

### 3.5 Predictors of attitude and competency of ED nurses

The model that included all predictors with attitude as a dependent variable (F = 1.68; P = 0.140; R^2^ = 0.027; adjusted R^2^ = 0.011) explained only 2.7% of the variance of attitude, where only educational level was found associated with attitude (t = −2.0; P = 0.047) after adjusting other variables in the model (Table 6). Another regression model with competency as a dependent variable and all other factors as independent variables (F = 6.0; P = 0.001; R^2^ = 0.09; adjusted R^2^ = 0.08) explained only  9.0% of the variance in competency where gender (t = −3.83; P = 0.001) was found associated with competency after adjusting other variables education (t = −1.42; P = 0.16), experience (t = −1.09; P = 0.28), age (t = −0.66; P = 0.51), facility (t = 1.15; P = 0.25; Table 7).

## 4. DISCUSSION

Generally, the attitudes of the ED nurses were predominantly positive, with their perceived competency levels falling below average. These findings provide concrete evidence for implementing changes in continuing education for ED nurses to enhance their competency in caring for mentally ill patients. This can influence positive clinical practice and improve patients’ quality of life.

ED is the primary place available to receive care and treatment during a mental health crisis. Generally, EDs are overwhelmed with extensive waiting times, a lack of inpatient beds, protracted delays in transferring patients to other inpatient facilities, overcrowding, and delays in treatment.^22^ The environment/resource, including limited appropriate physical space, time constraints, and overcrowding, insufficient knowledge and education regarding psychiatric illness, negative attitudes, and avoidance, can affect the experience of psychiatric patients.^23^ Physical and non-physical aggression and mental abuse are more common in EDs compared to mental health facilities. However, nurses working in psychiatric settings are well-trained to manage patients’ aggressive behavior and have better coping mechanisms compared with other healthcare settings.^24^ The possible explanation for these phenomena is that the prevalence of aggression is higher in EDs compared to other departments, and essential training for the management of aggression might be lacking in ED settings. An integrative review revealed that the care of mental health patients in the ED is affected by several factors: the lack of preparedness of ED nurses, insufficient education and training in aggression management, inadequate awareness of environmental safety, poor physical environment, and inadequate staffing and resources.^5^ Furthermore, a delay in assisting can lead to increasingly hostile behavior, beginning with frustration and angry grimaces, and potentially ending in anger due to the perceived lack of information.^25^

A recent study in Saudi Arabia supports that a positive attitude is crucial for promoting a healthy lifestyle, receiving appropriate and timely health services without any discrimination, throughout admission to the hospital.^9^ The present study findings support that the nurses have a positive attitude towards the mentally ill patients, which is in line with the previous studies.^7^^,^^9^ A study from the US indicated that mental health nurses exhibit a more positive attitude than medical-surgical nurses, indicating lower levels of stigma towards mental health patients.^26^ Interestingly, the participants consented that the mentally ill should not be considered inferior, subjected to discrimination, or a threat to society. This positive attitude is probably due to their high level of knowledge on the impact of stigmatization in the treatment, care, and recovery of mental health patients.^24^

Our findings indicate that ED nurses generally hold positive attitudes toward patients with mental illnesses. The attitude of ED nurses can be influenced by many factors. In the current study, attitude is not associated with any of the demographic variables. There was no consistent correlation identified between attitudes and perceived competency of the ED nurses. Additionally, the current study shows no association between attitude and other factors such as nursing specialty, experience in handling aggressive patients, training received in the MAB, suicide prevention, behavioral therapy, or mental status assessment. Whereas a previous study reported that personal or family/friend experiences with mental illness, younger age, lower education levels, and a lack of experience were found to be associated with stigma.^26^ Another study supported that long working experience has been associated with a positive attitude,^27^ whereas the current study failed to find an association with experience. Nurses with long periods of experience have a wide range of exposure to interacting with mentally ill patients. This potentially helps them to dispel the stigmatizing attitude towards such patients.^27^

The current study shows that ED nurses perceived themselves as average level of competence in caring for mentally ill patients, in line with the previous study.^28^ Also, this study highlighted specific areas for improvement of nurses’ competency, such as distinguishing between delirium and dementia, managing conflict with the patients, intervening with patients having hallucinations, and recommending psychotropic drugs to physicians, which is aligned with a previous study.^28^ The anticipated explanation is that ED nurses perceive constraints in their scope of practice and the organizational policies that do not mandate or facilitate nurses’ active participation in patient care decision-making processes. Handling challenging behavior in emergency settings requires a combination of skills, including ethical decision-making, professional communication skills, critical thinking ability, escalating behaviors, de-escalation techniques, and competence.^29^

No significant differences in BHCC subscale scores were found based on ED nurses, education status, experience in nursing, various training status, and prior psychiatric experience. Whereas young nurses, males, and those employed in certain hospital settings demonstrated significantly higher levels of perceived competence compared to their peers. This indicated that educational status, experience, previous experience in a psychiatric facility, and in-service training may not be the primary determinant of their competency. Whereas, young nurses, males, and working hospital may influence the nurses to sharpen their competency. This proves that organizational culture and personal attributes may be the primary determinants of their competency. The administration should prioritize strategies aimed at fostering a supportive working culture in the organization to improve the competency of ED nurses. Winokur et al.^28^ revealed that factors such as the nurses’ roles, shift work, previous psychiatric experience, and specialty training had significantly influenced the nurses’ competency levels. Additionally, higher scores in behavioral competence were associated with lower stigma and higher performance levels. Moreover, Nurses who had a history of workplace violence reported higher BHCC scores and individual work performance scores, which indicate behaviors related to teamwork, effort, and cooperation in their work environments.^30^ Given the finding that ED nurses demonstrate positive attitudes but average levels of perceived competency. The ED nurses may require a resource to support their essential training to advance their competencies. Further research is needed to focus on identifying the most effective ways to improve the ED nurse’s competency for caring for patients with mental illness and promote patient care and outcomes.

## 5. STRENGTHS AND LIMITATIONS

This study represents a pioneering effort to explore the attitudes and competencies of ED nurses in Qatar towards caring for mentally ill patients. The study enrolled representatives from four major EDs within the corporation. Therefore, the findings from this study provide a comprehensive overview that could accurately reflect the real picture and views of the ED nurses in Qatar. The study relies on online, self-administered surveys conducted through the enumerative sampling method. This introduces limitations in the generalization of the findings due to the non-probability sampling method. Additionally, the sensitive nature of the topic poses a significant challenge that could influence the responses of the participants.

### 5.1 Implications for practice

This study sheds light on the critical roles of ED nurses’ perceived competency and attitudes in the provision of care to psychiatric patients, which can significantly affect patients’ clinical outcomes and the overall quality of care within the ED. The attitudes of ED nurses not only influence the immediacy and quality of care received by psychiatric patients but also their overall experience in the care received from the ED. Other significant findings of this study reveal a competency domain below the desired level, signaling a clear call for the implementation of outcome-based mentorship programs specifically tailored to meet the unique demands of psychiatric patient care in the ED. These programs focus on enhancing practical skills, fostering critical thinking, and encouraging the application of evidence-based practices in nursing care.

## 6. CONCLUSION

Nurses working in non-psychiatric units often experience a lack of confidence and experience stigma when caring for patients with mental illnesses. This study sheds light on the critical need for targeted interventions. To overcome these challenges, it’s imperative to develop and implement resources specifically designed to improve competency and uplift the attitude of ED nurses. Such interventions should be targeted to minimize the stigma associated with mental illness. Implementing a peer support culture and providing easy access to mental health consultations for professional support can also play a vital role in building their confidence and competence.

## CONFLICT OF INTEREST

The authors have no conflict of interest to declare.

## PERMISSION TO REPRODUCE MATERIAL FROM OTHER SOURCES

The study used two validated questionnaires: The Opening Minds Scale for Health Care Providers (OMS-HC-15) and Behavioral Health Care Competency (BHCC), after obtaining permission from the authors.

## ACKNOWLEDGEMENTS

The authors would like to thank the study participants and the administration of all hospitals for their support.

## DATA AVAILABILITY STATEMENT

The data of the findings of the present study are available upon reasonable request to the corresponding author.

## AUTHORS CONTRIBUTIONS

HM provided a literature search, survey, data collection (survey), and manuscript writing. NH provided a literature search, survey, data analysis, and manuscript writing. RS provided data analysis, manuscript writing, peer review, and final approval. BC, AA, GS provided literature search, data collection (survey), manuscript writing, and peer review. All the authors have read and approved the final manuscript.

## Figures and Tables

**Table 1.** Demographic characteristics and clinical training of the participants.

Variables	Categories	Frequency % (*n* = 310)
Age	21–30 years	11 (3.55 %)
	31–40 years	224 (72.26%)
	41–50 years	56 (18.06 %)
	>51 years	19 (6.13%)
Gender	Male	183 (59.03 %)
	Female	127 (40.97 %)
Qualification	Diploma Nursing	23 (7. 42%)
	BSN	264 (85.16%)
	Master’s degree	23 (7.42%)
Experience	<5 years	96 (30.97%)
	6–10 years	90 (29.03%)
	≥11 years	124 (40.00%)
Facility	A	31 (10.00%)
	B	52 (16.77%)
	C	197 (63.55%)
	D	30 (9.68%)
Experience in the psychiatric unit	Yes	96 (30.97 %)
	No	214 (69.03%)
Experience in caring for aggressive patients	Yes	250 (80.65 %)
	No	60 (19.35%)
Trained in MAB	Yes	142 (45.81%)
	No	168 (54.19%)
Trained in suicide prevention	Yes	50 (16.13%)
	No	260 (83.87%)
Trained in behavioral therapy	Yes	49 (15.81%)
	No	261 (84.19%)
Trained in mental status assessment	Yes	94 (30.32%)
	No	216 (69.68%)

**Table 2.** The attitude and competency of the participants toward patients with mental illness.

Variable	Number of items	Total responses	Mean % ± SD %
Attitude	6	310	−14.16 ± 36.26
Competency	23	310	35.07 ± 25.73
Assessment	9	310	39.55 ± 26.02
Practice	8	310	30.40 ± 32.18
Recommended	2	310	12.74 ± 46.37
Resources	4	310	45.52 ± 29.99

**Table 3.** Factors associated with attitude and competency of ED nurses towards patients with mental illness.

Variables		Attitude	BHC competency	Assessment	Practice/intervention	Recommend psychotropics	Resource adequacy
	*N*	Mean (SD)					
Age							
21–30 years	11	0.106 (0.555)	0.502 (0.286)	0.530 (0.339)	0.489 (0.356)	0.250 (0.354)	0.591 (0.238)
31–40 years	224	−0.169 (0.354)	0.363 (0.246)	0.401 (0.252)	0.325 (0.298)	0.143 (0.459)	0.463 (0.302)
41–50 years	56	−0.092 (0.329)	0.303 (0.288)	0.375 (0.286)	0.214 (0.367)	0.112 (0.469)	0.415 (0.318)
>51 years	19	−0.114 (0.382)	0.263 (0.240)	0.319 (0.211)	0.214 (0.372)	−0.079 (0.534)	0.408 (0.228)
*P* value		0.056	0.038	0.16	0.015	0.18	0.27
Gender							
Female	127	−0.154 (0.353)	0.270 (0.251)	0.340 (0.245)	0.198 (0.331)	−0.021 (0.482)	0.403 (0.329)
Male	183	−0.133 (0.369)	0.406 (0.247)	0.434 (0.264)	0.377 (0.293)	0.231 (0.421)	0.492 (0.272)
*P* value		0.80	0.17	0.66	0.002	0.033	0.004
Qualification							
Diploma	23	−0.039 (0.388)	0.297 (0.326)	0.388 (0.323)	0.198 (0.388)	0.054 (0.470)	0.413 (0.342)
BSN	264	−0.142 (0.356)	0.363 (0.244)	0.403 (0.250)	0.320 (0.302)	0.139 (0.463)	0.469 (0.286)
Master’s degree	23	−0.239 (0.397)	0.262 (0.311)	0.306 (0.298)	0.225 (0.435)	0.065 (0.472)	0.331 (0.378)
*P* value		0.17	0.11	0.22	0.10	0.56	0.08
Experience							
<5 years	96	−0.185 (0.381)	0.392 (0.233)	0.423 (0.242)	0.365 (0.291)	0.126 (0.458)	0.512 (0.288)
6–10 years	90	−0.076 (0.364)	0.356 (0.239)	0.412 (0.242)	0.311 (0.293)	0.116 (0.457)	0.443 (0.295)
>11 years	124	−0.155 (0.342)	0.309 (0.278)	0.357 (0.278)	0.247 (0.352)	0.129 (0.471)	0.418 (0.307)
*P* value		0.12	0.055	0.12	0.025	0.98	0.06
Facility							
Facility A	31	−0.078 (0.360)	0.284 (0.296)	0.317 (0.302)	0.264 (0.376)	−0.016 (0.535)	0.403 (0.303)
Facility B	52	−0.118 (0.362)	0.268 (0.257)	0.326 (0.258)	0.218 (0.333)	0.043 (0.447)	0.350 (0.336)
Facility C	197	−0.143 (0.363)	0.383 (0.233)	0.425 (0.238)	0.330 (0.291)	0.181 (0.445)	0.493 (0.281)
Facility D	30	−0.233 (0.360)	0.347 (0.323)	0.398 (0.321)	0.316 (0.410)	0.066 (0.491)	0.437 (0.309)
*P* value		0.37	0.014	0.028	0.13	0.048	0.014

*P* values without significance values have been rounded to two decimals.

**Table 4.** The association between various training courses, competency, and the attitude of the participants.

		Attitude		BHC competency	
Variables	*n*	Mean	SD	Mean	SD
Specialty experience					
No	214	−0.134	0.337	0.314	0.256
Yes	96	−0.160	0.416	0.432	0.242
*P* value		0.013		0.37	
Experience in handling aggressive patients					
No	60	−0.125	0.332	0.242	0.286
Yes	250	−0.146	0.370	0.377	0.244
*P* value		0.17		0.05	
Management of assaultive behavior					
No	168	−0.135	0.336	0.333	0.251
Yes	142	−0.150	0.393	0.372	0.264
*P* value		0.046		0.72	
Suicide prevention					
No	260	−0.153	0.357	0.339	0.255
Yes	50	−0.082	0.387	0.413	0.262
*P* value		0.99		0.85	
Behavioral therapy					
No	261	−0.144	0.365	0.342	0.248
Yes	49	−0.131	0.355	0.397	0.300
*P* value		0.33		0.66	
Mental status assessment					
No	216	−0.145	0.344	0.316	0.248
Yes	94	−0.135	0.404	0.430	0.262
*P* value		0.27		0.42	

*P*-values without significant values have been rounded to two decimals.

**Table 5.** Correlation between attitude and competence of the ED nurses.

		Attitude	Competency
Attitude	Pearson correlation	1	−0.065
	Significance (2-tailed)		0.25
Competency	Pearson correlation	−0.065	1
	Significance (2-tailed)	0.25	

**Table 6.** Multivariable linear regression analysis for predictors of the attitude of ED nurses.

					95% Confidence interval	
Predictors	Coefficient	SE	t-test	P Value	Lower	Upper
Gender	−0.109	0.091	−1.20	0.231	−0.288	0.070
Education	−0.216	0.108	−2.00	0.047	−0.429	−0.003
Experience	0.049	0.064	0.77	0.442	−0.076	0.175
Age	−0.006	0.009	−0.67	0.503	−0.024	0.012
Facility	−0.106	0.055	−1.91	0.056	−0.215	0.003

**Table 7.** Multivariable linear regression analysis for predictors of competency of ED nurses.

					95% Confidence interval	
Predictors	Coefficient	SE	t-test	P Value	Lower	Upper
Gender	−0.239	0.062	−3.83	0.000	−0.361	−0.116
Education	−0.106	0.074	−1.42	0.156	−0.252	0.040
Experience	−0.048	0.044	−1.09	0.276	−0.134	0.038
Age	−0.004	0.006	−0.66	0.509	−0.017	0.008
Facility	0.044	0.038	1.15	0.252	−0.031	0.119
